# Beyond Bioenergetics: Emerging Roles of Mitochondrial Fatty Acid Oxidation in Stress Response and Aging

**DOI:** 10.3390/cells14241956

**Published:** 2025-12-09

**Authors:** Surim Bang, So-Hyun Choi, Seung Min Jeong

**Affiliations:** 1 Department of Biochemistry, College of Medicine, The Catholic University of Korea, Seoul 06591, Republic of Korea; 2 Department of Medical Science, College of Medicine, The Catholic University of Korea, Seoul 06591, Republic of Korea; 3 Institute for Aging and Metabolic Diseases, College of Medicine, The Catholic University of Korea, Seoul 06591, Republic of Korea

**Keywords:** fatty acid oxidation, stress response, age-related diseases, redox homeostasis, acetylation

## Abstract

Mitochondrial fatty acid oxidation (FAO) has long been recognized as a central pathway for energy production, providing acetyl-CoA, NADH, and FADH_2_ to sustain cellular growth and survival. However, recent advances have revealed that FAO exerts far broader roles beyond bioenergetics. FAO contributes to redox balance by generating NADPH for antioxidant defense, regulates protein acetylation through acetyl-CoA availability, and modulates stress signaling pathways to support cellular adaptation under nutrient or genotoxic stress. These emerging insights establish FAO as a metabolic hub that integrates energy homeostasis with redox regulation, epigenetic modification, and stress responses. Dysregulation of FAO has been increasingly implicated in aging and diverse pathologies, including cellular senescence, obesity, cancer and fibrosis. In this review, we highlight recent findings and provide an updated perspective on the expanding roles of mitochondrial FAO in stress responses and aging, with particular emphasis on its potential as a therapeutic target in age-associated diseases.

## 1. Introduction

Cells are constantly exposed to various internal and external stresses, including oxygen shortage, oxidative stress, nutrient starvation and DNA damage. To counteract these threats and maintain cellular homeostasis, cells have developed well-coordinated stress response pathways that range from the activation of repair and adaptation mechanisms to the induction of cell cycle arrest or programmed cell death [1]. The importance of stress response pathways is underscored by the fact that numerous human pathologies, such as neurodegenerative diseases, metabolic disorders, cancers, and aging, are frequently driven by defects in these critical cellular stress responses [2,3].

Growing evidence indicates that cellular metabolism plays a critical role in determining cell fate decisions and orchestrating adaptive responses to stress [4,5]. For example, it has been shown that DNA damage causes cells to block the entry of glutamine into the mitochondrial tricarboxylic acid (TCA) cycle, which is required for the proper DNA damage responses, including cell cycle arrest and DNA repair [6]. Disruption of this metabolic response leads to genomic instability and tumorigenesis. These results imply that metabolic regulation is essential for maintaining cellular integrity and suggest that there are distinct classes of stress response pathways governed by metabolic control.

Fatty acids (FAs) are among the major energy substrates for cells and organisms, particularly during fasting or periods of increased metabolic demand such as exercise. They can be stored as triglycerides (TGs) in adipose tissues between meals or oxidized to generate energy through mitochondrial fatty acid β-oxidation (FAO) [7]. Mitochondrial FAO plays a central role in maintaining cellular and systemic energy homeostasis by providing acetyl-CoA to the TCA cycle and supporting ATP production via oxidative phosphorylation (OXPHOS) [8]. In the liver, acetyl-CoA generated from FAO is also used for ketogenesis, leading to the production of ketone bodies that serve as alternative energy sources for peripheral tissues during prolonged fasting. Consequently, defects in FAO are often associated with multi-organ failure and metabolic disorders [7,9].

Although the essential role of FAO in energy production has been extensively studied, its functions in cellular stress responses remain poorly understood. This review summarizes current insights into the involvement of FAO in stress response pathways and highlights potential interventions targeting FAO as promising strategies to prevent or treat aging and age-associated diseases.

## 2. Current Understanding of Mitochondrial FAO

### 2.1. Fatty Acid Metabolism

The major lipids in the human diet are TGs, which consist of a glycerol backbone esterified with three FAs. Dietary TGs are digested in the small intestine by pancreatic enzymes, including pancreatic lipase, producing free FAs and 2-monoacylglycerol [10]. These FAs are re-esterified into TGs within chylomicrons, which then enter the bloodstream and transport dietary lipids to peripheral tissues. In addition to dietary sources, FAs can be synthesized de novo in the liver and adipose tissue when caloric intake exceeds energy expenditure [11]. The newly synthesized FAs are packaged into very-low-density lipoproteins (VLDLs) as a form of TGs, which are then secreted into the circulation [11]. TGs in both chylomicrons and VLDLs are hydrolyzed into FAs by lipoprotein lipase, an enzyme anchored to the luminal surface of endothelial cells. The liberated FAs are either stored in adipose tissue as TGs or oxidized in peripheral tissues such as skeletal muscle [12].

**Figure 1:**
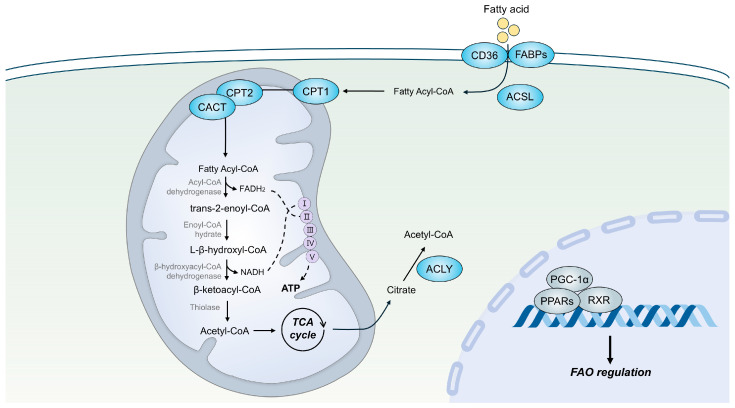
Overview of mitochondrial fatty acid oxidation (FAO). Fatty acids (FAs) enter cells through transporters such as CD36 and fatty acid-binding proteins (FABPs) and are activated to acyl-CoA by acyl-CoA synthetases (ACSLs). Before oxidation, the carnitine shuttle transfers fatty acyl-CoAs into mitochondria via carnitine palmitoyltransferase-1 (CPT1), carnitine-acylcarnitine translocase and CPT2. In the mitochondrial matrix, β-oxidation sequentially degrades fatty acyl-CoA through four recurring enzymatic steps—dehydrogenation, hydration, oxidation, and thiolytic cleavage—producing acetyl-CoA, FADH_2_, and NADH. The generated acetyl-CoA fuels the tricarboxylic acid (TCA) cycle, while FADH_2_ and NADH drive oxidative phosphorylation (OXPHOS) to generate ATP. Acetyl-CoA can also be exported as citrate to the cytosol, where it contributes to lipid biosynthesis and protein acetylation through ATP-citrate lyase (ACLY).

Cellular uptake of FAs is mediated by specific transporter proteins such as CD36 and fatty acid-binding proteins (FABPs) [13]. Once inside the cell, FAs are activated to acyl-coenzyme A (acyl-CoA) by acyl-CoA synthetases (ACSLs) before entering mitochondrial FAO or lipid synthesis pathways [14]. Prior to FAO, the carnitine cycle is required for transporting fatty acyl-CoAs across the mitochondrial membranes [15]. Carnitine palmitoyltransferase 1 (CPT1), located on the outer mitochondrial membrane, converts fatty acyl-CoAs into fatty acylcarnitines [16]. These fatty acylcarnitines are then translocated across the inner mitochondrial membrane in exchange for free carnitine by carnitine-acylcarnitine translocase [15]. Within the mitochondrial matrix, fatty acylcarnitines are converted back to fatty acyl-CoAs by a second acyltransferase, carnitine palmitoyltransferase 2 (CPT2) [16]. Finally, the regenerated fatty acyl-CoAs are ready for mitochondrial β-oxidation (Figure 1).

### 2.2. The Mitochondrial Fatty Acid β-Oxidation Cycle

Within the mitochondrial matrix, fatty acyl-CoA is sequentially degraded by the β-oxidation spiral, which removes two-carbon units from the acyl chain in the form of acetyl-CoA [7]. The β-oxidation pathway consists of four recurring enzymatic steps [17] (Figure 1). First, fatty acyl-CoA is converted to trans-2-enoyl-CoA by acyl-CoA dehydrogenase, generating FADH_2_ through the transfer of electrons to FAD. Next, enoyl-CoA hydratase adds water to form L-β-hydroxyacyl-CoA, which is subsequently dehydrogenated to β-ketoacyl-CoA by β-hydroxyacyl-CoA dehydrogenase, producing NADH in the process. In the final step, β-keto acyl-CoA is cleaved by thiolase to release one molecule of acetyl-CoA. The shortened fatty acyl-CoA then re-enters the cycle for the next round of oxidation until fatty acyl-CoA is fully degraded to acetyl-CoA [17]. The acetyl-CoAs produced through β-oxidation fuel the TCA cycle or serve as substrates for ketogenesis, while the FADH_2_ and NADH generated are oxidized via the mitochondrial respiratory chain to produce ATP through OXPHOS [7]. Each step of β-oxidation is catalyzed by different chain-length-specific enzymes [17]. For example, long-chain FAs are initially processed by long-chain acyl-CoA dehydrogenase (LCAD). As the FA chain shortens through successive rounds of β-oxidation, LCAD is replaced by enzymes specific for medium- or short-chain FAs, such as medium-chain acyl-CoA dehydrogenase (MCAD) or short-chain acyl-CoA dehydrogenase (SCAD), respectively.

### 2.3. Regulation of Mitochondrial FAO

The regulation of mitochondrial FAO occurs through multiple mechanisms. One key regulatory mechanism is transcriptional control of FAO-related enzymes. Peroxisome proliferator-activated receptors (PPARs), including PPARα, PPARβ/δ, and PPARγ, function as central transcriptional regulators of fatty acid metabolism [18,19]. During fasting or periods of increased energy demand, activated PPARs form heterodimers with retinoid X receptors (RXRs) to induce the expression of genes involved in FAO [19,20]. Another important transcriptional regulator of FAO is PPARγ coactivator-1α (PGC-1α), a transcriptional coactivator that enhances lipid metabolism by upregulating FAO-associated genes [21] (Figure 1).

**Figure 2:**
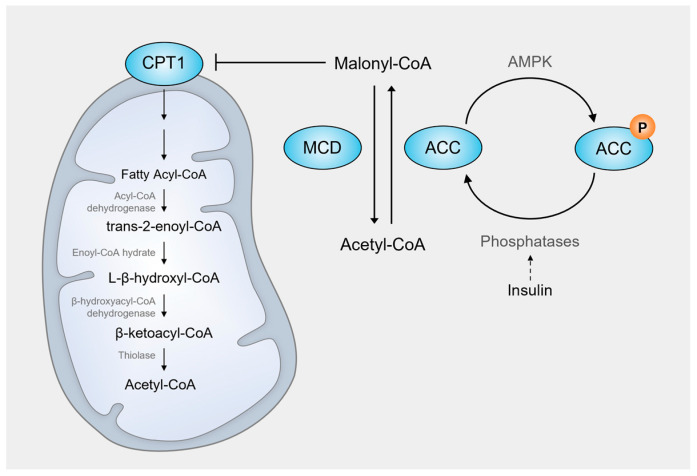
Regulation of mitochondrial FAO. CPT1 is the rate-limiting enzyme of mitochondrial FAO, and its activity is tightly controlled by malonyl-CoA, a potent CPT1 inhibitor synthesized from acetyl-CoA by acetyl-CoA carboxylase (ACC). Under low-energy conditions, AMP-activated protein kinase (AMPK) phosphorylates and inhibits ACC, thereby lowering malonyl-CoA levels and relieving CPT1 inhibition to promote FAO. Conversely, in energy-rich states, insulin activates phosphatases that dephosphorylate and stimulate ACC, increasing malonyl-CoA and suppressing FAO while favoring fatty acid synthesis. Malonyl-CoA decarboxylase (MCD), whose expression is induced by PPARs, also contributes to FAO regulation by converting malonyl-CoA back to acetyl-CoA, thus further enhancing CPT1 activity and FAO flux.

An additional key regulatory mechanism of FAO involves CPT1, the rate-limiting enzyme for mitochondrial FAO, and its activity is a crucial determinant of FAO [16] (Figure 2). CPT1 activity is tightly regulated by malonyl-CoA, which is synthesized from acetyl-CoA by acetyl-CoA carboxylase (ACC). Under low-energy conditions, AMP-activated protein kinase (AMPK) becomes activated and phosphorylates ACC, leading to its inhibition. As a result, malonyl-CoA levels decrease, thereby relieving CPT1 inhibition and promoting FAO [22]. Cellular malonyl-CoA levels are also regulated by malonyl-CoA decarboxylase (MCD); its expression is transcriptionally induced by PPARs. Increased MCD expression converts malonyl-CoA to acetyl-CoA, reduces intracellular malonyl-CoA levels, and consequently enhances CPT1 activity [23]. In the liver, ACC is also regulated by hormone-dependent mechanisms. Under energy-rich conditions, elevated insulin levels stimulate phosphatases that dephosphorylate and activate ACC [24]. A high insulin/glucagon ratio further promotes ACC expression. As a result, malonyl-CoA levels increase, leading to the inhibition of CPT1 [25]. Thus, in the fed state, elevated malonyl-CoA suppresses FAO and instead promotes fatty acid synthesis in the liver.

### 2.4. Canonical Role of Mitochondrial FAO

As FAs are a major fuel source for cells and organisms, the primary role of FAO is energy production. Mitochondrial FAO is particularly important in energy-demanding organs such as skeletal muscle, kidney, and heart [8]. In response to increased metabolic need, FAs released from TG stores in adipose tissue are transported to muscle, where they are catabolized via FAO. The resulting FADH_2_ and NADH are utilized to generate ATP, while Acetyl-CoA enters the TCA cycle to refill mitochondrial carbon pool. Notably, metabolically active tissues such as the heart and kidneys preferentially rely on FAO to meet their high and continuous energy demands [17]. In the liver, acetyl-CoA derived from FAO is also used for the synthesis of ketone bodies, including acetoacetate and β-hydroxybutyrate [26].

The essential role of mitochondrial FAO in energy homeostasis is highlighted in patients with FAO disorders. Among 25 enzymes and transporters involved in FAO, at least 15 are affected by autosomal recessive inheritance defects [7]. For example, MCAD deficiency, caused by a single base substitution, is one of the most common inherited disorders in metabolic disorders [27]. Patients with FAO deficiencies often suffer from heart failure, skeletal muscle diseases and metabolic decompensation [28]. The most frequent symptom of such patients is intermittent hypoketotic hypoglycemia during fasting [7,28], which can be life-threatening without early diagnosis and intervention.

## 3. Beyond Bioenergetics: Mitochondrial FAO in Cellular Stress and Aging

### 3.1. FAO as a Metabolic Integrator of Cellular Stress Responses

**Figure 3:**
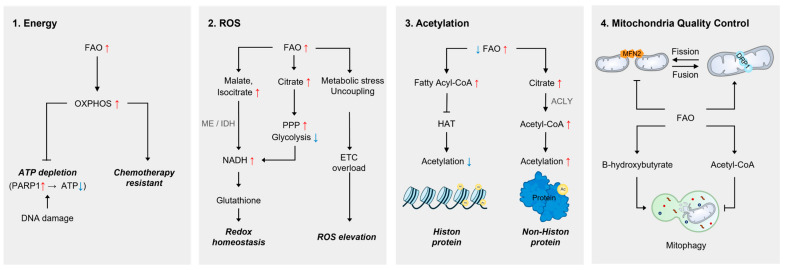
Functional roles of mitochondrial FAO in cellular stress adaptation. Mitochondrial FAO supports cellular adaptation to stress through four integrated mechanisms. (1) Energy production: FAO-derived acetyl-CoA fuels the TCA cycle and OXPHOS to generate ATP, compensating for energy loss following PARP1-mediated ATP depletion after DNA damage. Enhanced FAO and OXPHOS sustain the metabolic demands of stressed or chemotherapy-resistant cancer cells. (2) Redox regulation: FAO contributes to antioxidant defense by generating reducing equivalents via malic enzyme and isocitrate dehydrogenase, while FAO-derived citrate redirects glucose toward the pentose phosphate pathway to boost NADPH production and support glutathione-mediated ROS detoxification. FAO inhibition impairs NADPH regeneration and triggers oxidative stress, whereas excessive FAO flux can also elevate mitochondrial ROS production. (3) Protein acetylation: FAO-derived acetyl-CoA replenishes mitochondrial and cytosolic acetyl-CoA pools, sustaining acetylation of metabolic and stress-response proteins through ACLY. Accumulated fatty acyl-CoAs can inhibit histone acetyltransferase (HAT) activity, linking FAO to epigenetic and transcriptional regulation under stress. (4) Mitochondrial quality control: FAO interfaces with mitochondrial dynamics and mitophagy. FAO activation promotes DRP1-dependent fission to accommodate higher β-oxidation flux, whereas reduced FAO favors MFN2-mediated fusion and lipid storage. FAO-derived metabolites, including β-hydroxybutyrate and acetyl-CoA, modulate PINK1/Parkin-dependent mitophagy and autophagy-related protein acetylation. Collectively, these mechanisms position FAO as a central metabolic hub integrating energy, redox balance, epigenetic regulation, and organelle quality control during cellular stress adaptation.

In the classical view, the primary function of mitochondrial FAO is to maintain cellular energy homeostasis. However, accruing evidence suggests that FAO serves a broader role as a metabolic integrator of cellular stress responses. This emerging perspective is supported by recent studies demonstrating that FAO is regulated in response to cellular stressors such as hypoxia and DNA damage [29,30,31]. In this section, we survey the emerging mechanisms through which FAO interfaces with metabolic and organelle signaling networks to support cellular adaptation (Figure 3).

#### 3.1.1. Energy

Most biochemical reactions require energy. Because cellular stress pathways are proactive and energy-intensive, it has been proposed that FAO contributes to stress responses by serving as an important energy source. Indeed, both acute and chronic genotoxic stresses have been shown to activate mitochondrial FAO [32]. This FAO upregulation could support the bioenergetic needs of stressed cells, in part by compensating for ATP depletion caused by PARP-1 activation following DNA damage [33]. In this regard, chemotherapy-resistant acute myeloid leukemia (AML) cells exhibit increased expression of FA transporter CD36, along with enhanced FAO and OXPHOS [34]. This study also showed that targeting FAO induces a metabolic shift toward reduced OXPHOS and sensitizes cancer cells to chemotherapeutic agents [34]. Although the precise molecular mechanisms by which FAO-derived energy production contributes to drug resistance remain unclear, these findings suggest that FAO plays a key role in maintaining energy homeostasis under stressed conditions and may facilitate cellular adaptation.

#### 3.1.2. Reactive Oxygen Species (ROS)

Reactive oxygen species (ROS), mainly generated by mitochondria, have been recognized as crucial modulators of numerous cellular signaling pathways, including those involved in stress response pathways [35,36]. Various forms of cellular stress often disrupt redox homeostasis, leading to impaired signaling cascades and exacerbated cellular damage [37]. Therefore, it is essential for cells to monitor and regulate ROS levels in response to fluctuations in redox status. At the same time, maintaining adequate reducing power is critical for preventing oxidative stress and ensuring cell survival under stress conditions.

Although glucose and glutamine are widely recognized as the two most important nutrients for regulating intracellular ROS levels [38,39], FAO also plays a significant role in maintaining redox homeostasis. FAO provides reducing equivalents in the form of NADPH, which are essential for the synthesis and regeneration of glutathione, a major intracellular antioxidant [40]. For example, FAO elevates intracellular malate and isocitrate pools, which serve as substrates for NADPH production through malic enzyme or isocitrate dehydrogenase [41]. Moreover, FAO-derived citrate inhibits phosphofructokinase 1 (PFK1), thereby redirecting glucose from glycolysis to the pentose phosphate pathway (PPP), which further promotes NADPH synthesis [42]. The relevance of mitochondrial FAO in ROS regulation has been supported by several findings. In glioma cells, FAO inhibition leads to a marked reduction in cellular NADPH levels, resulting in oxidative stress-induced cell death [43]. Similarly, it has been shown that endothelial cells rely on FAO to maintain redox balance and resist oxidative stress-mediated dysfunction [44].

FAO contributes to antioxidant defense by supplying NADPH and supporting glutathione-dependent detoxification, but it is also intrinsically an oxidative mitochondrial process. Enhanced FAO increases electron flow through the electron transport chain, which can elevate mitochondrial ROS production, particularly under conditions of metabolic stress, ETC overload, or uncoupling [45,46,47]. Thus, FAO exerts a dual influence on redox biology: it provides reducing power that supports antioxidant capacity, yet can also serve as a source of ROS that drives oxidative damage when mitochondrial handling of electron flux is compromised. While the precise role of FAO-dependent ROS regulation in response to specific cellular stress remains to be elucidated, these findings suggest that FAO provides critical reducing power necessary to preserve cellular redox homeostasis and support stress response pathways.

#### 3.1.3. Acetylation

To rapidly respond to cellular stress, many proteins involved in stress response pathways are activated by posttranslational modifications such as phosphorylation and acetylation [48]. Because intracellular acetyl-CoA levels are critical determinants of protein acetylation [49,50] and FAO is one of the major sources of acetyl-CoA, mitochondrial FAO has emerged as a key regulator of cellular acetylation dynamics. Palmitate, the most abundant saturated FA in human cells, undergoes seven rounds of mitochondrial β-oxidation to generate eight molecules of acetyl-CoA. These acetyl-CoA molecules not only replenish the mitochondrial acetyl-CoA pool but are also exported to the cytosol in the form of citrate. Citrate, transported via mitochondrial citrate carrier, can be converted back to acetyl-CoA by ATP-citrate lyase (ACLY) and contribute to the cytosolic acetyl-CoA pool [51]. In support of this notion, recent studies have provided compelling evidence that FAO-derived acetyl-CoA serves as a major carbon source for protein acetylation not only within mitochondria but also in the cytoplasm and nucleus [30,31,52].

While FAO-derived acetyl-CoA is an important determinant of protein acetylation, an additional mechanism has been proposed that is independent of acetyl-CoA levels and thus not directly affected by mitochondrial β-oxidation itself. Montgomery et al. demonstrated that fatty acyl-CoAs can directly inhibit histone acetyltransferase (HAT) activity by blocking its active site [53]. Indeed, enhanced fatty acyl-CoA synthesis following overexpression of ACSL was shown to decrease histone acetylation [53]. Since fatty acyl-CoA formation precedes mitochondrial FAO, defects in β-oxidation may lead to the intracellular accumulation of fatty acyl-CoAs. This implies that FAO could influence protein acetylation not only by supplying acetyl-CoA but also by modulating HAT activity through its upstream intermediates. Although it remains unclear to what extent this mechanism is applicable across diverse stress conditions, these findings suggest that FAO may regulate stress signaling pathways by modulating protein acetylation through multiple mechanisms.

#### 3.1.4. Mitochondrial Quality Control

Mitochondrial quality control, encompassing fusion–fission dynamics and mitophagy, is essential for cellular adaptation to stress and is tightly linked to metabolic state. FAO has been shown to interact with mitochondrial morphology [54]. Reduced FAO is associated with enhanced MFN2-mediated fusion, generating elongated mitochondrial networks that favor lipid storage rather than oxidation [55]. In contrast, FAO activation promotes DRP1-dependent fission, producing fragmented mitochondria that accommodate higher β-oxidation flux and facilitate efficient fatty acid utilization while preventing lipid accumulation [55,56]. FAO-derived metabolites may also influence mitophagy: the ketone body β-hydroxybutyrate enhances PINK1/Parkin-dependent mitophagy through AMPK–SIRT–PGC-1α signaling [57,58], and FAO-derived acetyl-CoA can regulate acetylation of autophagy-related proteins [29]. Although the mechanistic links remain to be fully defined, these findings suggest that FAO integrates mitochondrial dynamics and mitophagy to maintain organelle quality and cellular homeostasis during stress and aging.

### 3.2. Mitochondrial FAO in Aging and Aging-Linked Pathologies

**Figure 4:**
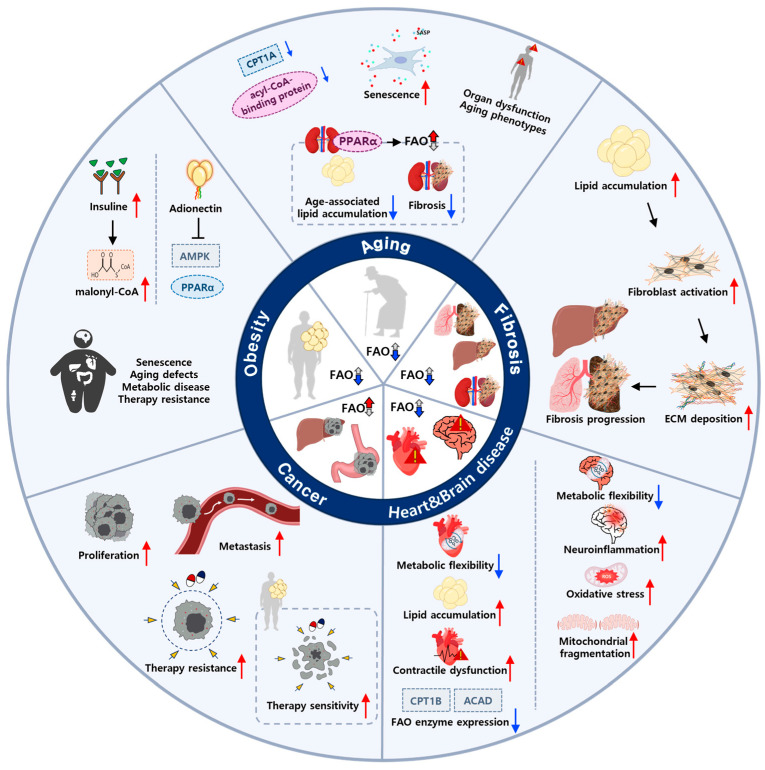
Involvement of mitochondrial FAO in aging and age-associated diseases. Mitochondrial FAO contributes to cellular and organismal homeostasis, and its dysregulation is increasingly recognized as a hallmark of aging-related pathologies. (1) Cellular senescence: Reduced FAO activity, including loss of CPT1C or acyl-CoA binding protein, promotes lipid accumulation, senescence induction, and the senescence-associated secretory phenotype (SASP). Restoration of FAO prevents senescence and preserves mitochondrial function. (2) Obesity: Chronic hyperinsulinemia elevates malonyl-CoA through ACC activation, inhibiting CPT1 and suppressing FAO, while decreased adiponectin lowers AMPK and PPARα activity. Impaired FAO contributes to metabolic stress, inflammation, and obesity-induced aging phenotypes. (3) Cancer: Many tumors reprogram FAO to meet high bioenergetic and redox demands. Enhanced FAO fuels OXPHOS, supports NADPH regeneration, and promotes metastasis and chemoresistance. Conversely, FAO inhibition sensitizes cancer cells to therapy. (4) Fibrosis: Diminished FAO in hepatic, renal, and cardiac tissues leads to lipid overload and activation of TGF-β-driven fibrogenic pathways. Pharmacological activation of PPARα or AMPK enhances FAO, suppresses myofibroblast differentiation, and alleviates fibrosis. (5) Cardiovascular and neurodegenerative diseases: Aging reduces FAO capacity in heart and brain, impairing metabolic flexibility. In the heart, diminished FAO leads to lipid accumulation, energetic deficits, and contractile dysfunction. In the brain, altered acylcarnitine profiles and reduced ketone utilization contribute to neuronal energy failure, oxidative stress, and neurodegeneration. Together, these examples illustrate that mitochondrial FAO serves as a metabolic checkpoint integrating energy metabolism with stress response, whose dysregulation accelerates aging and drives chronic disease progression.

While the essential role of mitochondrial FAO in human physiology, particularly during periods of increased fuel demand, has been well established through studies of patients with FAO disorders, recent findings have implicated FAO in the context of aging. Here, we summarize accumulating evidence linking FAO to multiple age-associated phenotypes and pathologies (Figure 4).

#### 3.2.1. Cellular Senescence and Mitochondrial Aging

Cellular senescence is one of the important cellular stress responses [59]. Upon various cellular stress stimuli, cells cease proliferation and enter a state of permanent cell cycle arrest, known as cellular senescence [60]. While senescence has some beneficial effects by limiting tumor development or accelerating wound healing, recent studies have shown that senescent cells progressively accumulate in multiple organs with age, and their removal can delay or attenuate chronic diseases and age-associated disorders [61]. Although the precise mechanisms by which mitochondrial FAO regulates cellular senescence remain under active investigation, numerous studies suggest that reduced FAO is a characteristic feature of senescent cells. For instance, decreased CPT1C expression has been observed in senescent pancreatic ductal adenocarcinoma cells, contributing to both senescence induction and the senescence-associated secretory phenotype (SASP) [62]. Similarly, loss of acyl-CoA-binding protein, which impairs FAO, has been shown to trigger senescence in glioblastoma cells [63]. Consistent with these observations, our recent study demonstrated that inhibition of FAO promotes senescence induction in human fibroblasts [64].

Dysregulation of FAO is implicated not only in cellular senescence but also in various age-related pathologies. For example, defective β-oxidation leads to the accumulation of fatty acyl-CoAs and acylcarnitines, which can drive lipotoxicity, mitochondrial dysfunction, and metabolic rigidity—key contributors to tissue aging and organ failure. Supporting this concept, metabolomic profiling of older adults with insomnia revealed significant elevations in medium- and long-chain acylcarnitines [65], indicating incomplete FAO and impaired mitochondrial metabolic flexibility. Consistently, hepatic senescence cells lose the ability to oxidize FAs, leading to lipid accumulation and hepatic dysfunction [66]. Additionally, several studies have demonstrated that FAO plays a protective role against age-related functional defects [67], as evidenced by PPARα-mediated induction of FAO, alleviating age-associated lipid accumulation and fibrosis in renal epithelial cells [68]. Similarly, induction of FAO by fasting has been reported to augment intestinal stem cell function in aged mice [69]. Thus, these findings further strengthen the significance of FAO in mitigating aging-related defects.

#### 3.2.2. Obesity-Driven Metabolic Aging

Obesity, characterized by excessive fat accumulation, is closely associated with various metabolic disorders such as type 2 diabetes, atherosclerosis and liver disease [70]. Abnormal fat accumulation in obesity can impair the proper regulation of mitochondrial FAO [31,71]. In obese individuals, chronically elevated blood insulin levels increase cellular malonyl-CoA levels via activation of ACC, thereby suppressing FAO through inhibition of CPT1 [72]. Additionally, adiponectin, a hormone primarily secreted from adipocytes, declines as adipose tissue mass expands [73]. Because adiponectin activates both AMPK and PPARα [73], its reduced secretion further contributes to the downregulation of FAO activity in obesity.

Accumulating evidence demonstrates that obesity is closely related to cellular senescence and age-related chronic diseases. Obesity accelerates multiple cellular aging processes, including telomere attrition, mitochondrial dysfunctions and altered nutrient sensing, while elimination of senescent cells can prevent obesity-induced inflammation and diabetes complications [74,75]. In this regard, Ogrodnik et al. reported that obesity-induced senescence in glial cells of mouse brain drives anxiety and reduces neurogenesis, which were alleviated by clearance of senescent cells [76]. Given that obesity disrupts proper FAO regulation and is associated with increased senescence and aging-related pathologies, it is plausible that impaired FAO contributes to the development of obesity-induced aging phenotypes. Therefore, targeting FAO may hold significant therapeutic potential for mitigating obesity-driven aging and related pathologies in obese individuals.

#### 3.2.3. Cancer Metabolic Reprogramming

Aging is considered one of the main risk factors for cancer development. Cancer cells are known to hijack metabolism to sustain their uncontrolled proliferation and ensure survival under various stress conditions [77,78]. Among these metabolic alterations, the differential use of FAs has emerged as a prominent metabolic feature in many cancer types [79,80]. In particular, mitochondrial FAO has been implicated in diverse aspects of cancer progression, including proliferation, metastasis and survival [40,81]. Because this topic has been extensively reviewed elsewhere, here we briefly summarize recent findings.

Many studies have demonstrated that mitochondrial FAO is critical for sustaining cancer cell proliferation, and that genetic or pharmacological inhibition of FAO dampens the unrestricted growth of cancer cells. For example, recent evidence indicates that the availability of fatty acyl-CoA for mitochondrial FAO acts as a primary regulator of glioblastoma multiforme cell proliferation [63]. FAO also contributes to cancer metastasis. In melanoma cells, FAO is required for metastatic spread of cancer cells to sentinel lymph nodes. Activation of yes-associated protein (YAP) by bile acid accumulation upregulates the expression of genes encoding FAO enzymes in metastatic cancer cells [82]. In this regard, FAO supports melanoma cell migration partly by regulating autophagosome formation [83]. Moreover, FAO plays a critical role in cancer cell survival under metabolically and oxidatively stressful conditions. By producing ATP during nutrient limitation and generating NADPH to counteract redox stress, FAO serves as a vital mechanism for cancer cells in challenging environments [84,85]. In particular, cancers exhibiting an OXPHOS gene expression signature, such as leukemia and large B cell lymphoma, show strong dependency on FAO for survival [86]. As mentioned earlier, FAO is a critical catabolic pathway fueling OXPHOS in acute AML cells, especially those resistant to chemotherapy [34]. Interestingly, recent studies have shown that FAO is not only important for sustaining cancer cell viability but is also required for proper cell death after DNA damage. Hwang et al. demonstrated that obesity-induced defects in FAO induction contribute to chemoresistance in obese mouse models [31].

Collectively, these findings highlight the multifaceted roles of mitochondrial FAO in cancer cell survival and progression, and further research will be required to fully understand the complex and diverse functions of FAO in cancer. In conclusion, mitochondrial FAO has emerged as an essential metabolic pathway contributing to various aspects of cancer cells, making it an attractive target for developing therapeutic interventions against diverse malignancies.

#### 3.2.4. Fibrotic Remodeling During Aging

Fibrosis is a hallmark of chronic tissue damage and aging, characterized by excessive extracellular matrix (ECM) deposition driven by activated myofibroblasts [87]. Emerging evidence indicates that mitochondrial FAO plays a pivotal role in regulating fibroblast activation and fibrogenesis. In healthy tissues, FAO supports energy homeostasis and prevents the pathological accumulation of intracellular lipids. However, impaired FAO, resulting from mitochondrial dysfunction or defects in FAO regulation, can lead to lipid accumulation, metabolic stress, and activation of pro-fibrotic pathways [68,88,89]. For example, decreased expression of CPT1A in hepatic stellate cells promotes lipid droplet accumulation, increased α-smooth muscle actin (α-SMA) expression, and enhanced ECM production [90]. Similarly, reduced expression of key FAO enzymes has been reported in renal fibrosis [91], and FAO inhibition has been shown to facilitate TGF-β–mediated fibrosis development [92].

Conversely, interventions that restore or enhance FAO have demonstrated anti-fibrotic effects across multiple organ systems. Pharmacological activation of PPARα or AMPK not only stimulates FAO but also suppresses TGF-β signaling and inhibits myofibroblast differentiation. In preclinical models, PPARα agonists have reduced collagen deposition in both hepatic and renal fibrosis [91,93], while AMPK activators have mitigated cardiac and pulmonary fibrosis by restoring mitochondrial function and lipid clearance [94,95]. Mechanistically, FAO enhancement may counteract fibrosis by reducing mitochondrial ROS production, replenishing acetyl-CoA pools to regulate gene expression via histone acetylation and maintaining NAD+/NADH balance. Collectively, these findings indicate that FAO is an important metabolic checkpoint in fibrotic reprogramming and suggest that targeting FAO could be a promising therapeutic approach for fibrosis-driven chronic diseases.

#### 3.2.5. Cardiovascular and Neurodegenerative Aging

Aging is a major risk factor for both cardiovascular and neurodegenerative diseases, and accumulating evidence indicates that impaired mitochondrial FAO contributes to dysfunction in both systems. In the heart, FAO provides 60–90% of ATP under physiological conditions, and aging-associated reductions in FAO capacity result in metabolic inflexibility, lipid accumulation, and contractile dysfunction, features observed in cardiac aging and heart failure [96,97]. Decreased expression of key FAO enzymes, including CPT1B and ACADs, has been reported in aged myocardium, while restoration of FAO through PPARα or AMPK activation improves cardiac energetics and attenuates pathological remodeling [98,99].

Similarly, aged brain exhibits a marked decline in mitochondrial metabolic flexibility, and recent studies have highlighted altered acylcarnitine profiles and reduced FAO capacity in neurodegenerative disorders such as Alzheimer’s and Parkinson’s disease [100,101,102]. FAO-derived ketone bodies serve as critical alternative fuels during aging, and impaired ketone utilization has been associated with neuronal energy deficits and cognitive decline [103,104]. Moreover, FAO dysregulation can exacerbate neuroinflammation, oxidative stress, and mitochondrial fragmentation, key drivers of neurodegeneration [105,106]. Together, these findings underscore the importance of maintaining FAO capacity to preserve cardiac and neuronal resilience during aging.

## 4. Conclusions and Future Prospects

There have been tremendous advances in our understanding of the diverse functions of mitochondrial FAO in various cellular processes. Growing evidence indicates that mitochondrial FAO is intimately involved in maintaining cellular homeostasis and coordinating stress responses. Nevertheless, the metabolic aspects of stress response remain incompletely explored, and the roles of FAO in coordinating nutrient and energy homeostasis, as well as cellular stress and adaptation pathways, are complex. Thus, further study should clarify how FAO not only supplies the energy required to operate stress response pathways but also scavenges ROS and modulates protein acetylation to facilitate cellular adaptation to stress. Moreover, although our knowledge of FAO’s roles in stress response and senescence at a cellular level is expanding, its contribution to organismal stress adaptation and aging remains to be elucidated. Since the elderly population is rapidly increasing, defining the physiological functions and regulatory mechanisms of mitochondrial FAO in stress and aging could open new avenues for treating age-associated disease and developing targeted therapeutics.

## Data Availability

No new data were created or analyzed in this study.
